# Predictive factors for successful limb salvage surgery in diabetic foot patients

**DOI:** 10.1186/1471-2482-14-113

**Published:** 2014-12-30

**Authors:** Matthew Seung Suk Choi, Seung Bae Jeon, Jang Hyun Lee

**Affiliations:** Department of Plastic and Reconstructive Surgery, Hanyang University Guri Hospital, College of Medicine, Hanyang University, 153 Gyeongchun-ro, Guri, 471-701 South Korea

**Keywords:** Diabetic foot, Major limb amputation, Limb salvage

## Abstract

**Background:**

The goal of salvage surgery in the diabetic foot is maximal preservation of the limb, but it is also important to resect unviable tissue sufficiently to avoid reamputation. This study aims to provide information on determining the optimal amputation level that allows preservation of as much limb length as possible without the risk of further reamputation by analyzing several predictive factors.

**Methods:**

Between April 2004 and July 2013, 154 patients underwent limb salvage surgery for distal diabetic foot gangrene. According to the final level of amputation, the patients were divided into two groups: Patients with primary success of the limb salvage, and patients that failed to heal after the primary limb salvage surgery. The factors predictive of success, including comorbidity, laboratory findings, and radiologic findings were evaluated by a retrospective chart review.

**Results:**

The mean age of the study population was 63.9 years, with a male-to-female ratio of approximately 2:1. The mean follow-up duration was 30 months. Statistical analysis showed that underlying renal disease, limited activity before surgery, a low hemoglobin level, a high white blood cell count, a high C-reactive protein level, and damage to two or more vessels on preoperative computed tomography (CT) angiogram were significantly associated with the success or failure of limb salvage. The five-year survival rate was 81.6% for the limb salvage success group and 36.4% for the limb salvage failure group.

**Conclusion:**

This study evaluated the factors predictive of the success of limb salvage surgery and identified indicators for preserving as much as possible of the leg of a patient with diabetic foot. This should help surgeons to establish the appropriate amputation level for a case of diabetic foot and help prevent consecutive operations.

## Background

Approximately 3–4% of diabetic patients develop foot ulcers sometime during their life. One of the most important strategies for the management of the diabetic foot is to prevent complications that may necessitate a major limb amputation. Even with appropriate treatment, some patients must undergo major amputation or a limb salvage operation [1, 2]. These operations are not only a huge emotional and social burden to the patients due to physical impairment, but also a financial burden [3, 4]. In recent decades, systemization of multidisciplinary management and implementation of free tissue transfer in diabetic foot treatment have led to a notable decrease in the major amputation rate [5, 6]. The key to limb salvage surgery is maximal retention of the limb and minimization of the amputation level. Free tissue transfer has become an alternative option to major amputation for elderly diabetic patients [7, 8]. Successful limb salvage, defined as a stump fit for functional ambulation, is mostly determined by the level of amputation. It is mostly affected by preservation of the talus and calcaneus because it minimizes limb length discrepancy and preserves the heel pad [9]. The level of Chopart amputation is the most proximal among lower limb amputation locations that preserve the talus and calcaneus. Although disputable, the Chopart amputation has been defined as the threshold of successful limb salvage [10–12]. The incidence of reamputation following first toe or transmetatarsal amputation associated with diabetes mellitus has been found to be nearly one-third. Nearly 40% of patients with diabetic foot who had amputations at the foot level have a history of previous amputation [13, 14]. Surgeons should preserve as much limb length as possible. However, it is also important to avoid reamputation, since it is a massive surgical burden to diabetic patients, who usually already are in a poor general condition and face financial difficulties.

The purpose of this article is to provide information on determining the optimal amputation level, preserving as much limb length as possible without requiring additional reamputation by analyzing several predictive factors.

## Methods

Approval for this retrospective study was obtained from the Institutional Review Board on Human Subjects Research and the Ethics Committee, Hanyang University Guri Hospital (IRB No. 2014–07-010). The study population was composed of patients who presented to the Department of Plastic Surgery with a diabetic foot complication from April 2004 to July 2013. The inclusion criterion was gangrene of the distal foot (distal to the metatarsophalangeal joint) that required hospitalization and amputation. Patients with complete healing without amputation were excluded from this study. The patients were divided into two groups based on their latest amputation level. The group with successful limb salvage consisted of patients with a preserved talus and calcaneus after amputation at the Chopart level or distal to it. The other group comprised patients in whom the limb could not be preserved. The patients of this group required reamputation more proximal than the Chopart level following an unsuccessful limb salvage operation.

The primary outcome measures included age, sex, smoking status, presence of comorbidities (hypertension, ischemic heart syndrome, stroke, chronic renal failure, chronic osteomyelitis), status of premorbid activities of daily living, preoperative laboratory findings, and preoperative radiologic findings. Preoperative laboratory investigations, including the hemoglobin level (Hb), white blood cell (WBC) count, glycosylated hemoglobin (HbA1c), creatinine, and C-reactive protein (CRP) levels were collected. A preoperative computed tomography (CT) angiogram was performed in all patients to evaluate the number of abnormal vessels and the state (patent, partial occlusion, total occlusion) of each vessel of the lower extremity. The secondary outcome measures were six-month and five-year survival rates. Kaplan-Meier survival estimate curves were calculated for all patients.

### Statistical analysis

All statistical analyses were performed using Stata/SE 12.0 with statistical significance set at P < 0.05. To determine the statistically significant differences between the two groups, an independent t-test was used for hemoglobin and a Mann–Whitney U test was used for numerical prognostic factors. Fisher’s exact test and logistic regression analysis were used for categorical prognostic factors. Kaplan-Meier survival estimate curves were also calculated, and the log-rank test was used to compare the survival rate.

## Results

### Patient profiles

Of the 461 consecutively admitted patients with diabetic foot complications between 2004 and 2013, 307 patients with complete remission without amputation were identified and excluded from the study. The other 154 patients, who underwent limb salvage surgery, and who were classified as grade 2–4 in the Wagner system, were divided into two groups. The group with successful limb salvage consisted of 124 patients, and the group with limb salvage failure consisted of 30 patients (Table 1).

**Table 1:** **Final amputation level of both groups**

	Success group	Failure group
Toe amputation or disarticulation	100	-
Ray (metatarsal)	8	-
Transmetatarsal	6	-
Midfoot Lisfranc	7	-
Chopart	3	-
Syme	-	-
BK amputation	-	30
Total	124	30

BK; below knee.

### Risk factor evaluation

We evaluated various factors related to the success and failure of limb salvage (Table 2). Statistical analysis showed that chronic renal failure and the activities of daily living were significantly associated with the success or failure of limb salvage. Our analysis found no significant association between the outcome of limb salvage and age, sex, smoking status, type of diabetes, ischemic heart syndrome, stroke, or hypertension. The outcome of limb salvage was significantly associated with Hb level, WBC count, and CRP level, but not with HbA1c. Osteomyelitis had no significant relationship with the outcome. On preoperative CT angiogram, multivariate analysis showed that the number of damaged vessels in the failure group was greater than that in the success group with statistical significance. The comparison between the lack of vessel damage and single vessel damage was not statistically significant. The comparison between damage to two or more vessels and less than two vessels showed that the failure group had damage to two or more vessels significantly more often. The failure group also contained a significantly greater proportion of cases with damage to three vessels. For each of the major vessels, cases in the failure group were significantly more likely to have an occlusion. The odds ratio was 10.405 for the anterior tibial artery, 5.062 for the posterior tibial artery, and 4.229 for the perineal artery, all with a significance level of P < 0.05.

**Table 2:** **Results of evaluation of the value of factors as predictive of outcome**

Factors		Outcome								
			Success group		Failure group		P-value	OR	95% CI	
			n (%)	Mean (SD)	n (%)	Mean (SD)			Lower limit	Upper limit
Age (years)							0.449	1.13	0.818	1.574
	≤40		4(3.2%)		0(0%)					
	41-50		23(18.6%)		4(13.3%)					
	51-60		22(17.7%)		3(10.0%)					
	61-70		30(24.2%)		13(43.3%)					
	71-80		38(30.7%)		10(33.3%)					
	>80		7(5.7%)		0(0%)					
	Total		124(100%)		30(100%)					
Sex							0.282			
	Male		81(65.3%)		23(76.7%)					
	Female		43(34.7%)		7(23.3%)					
Smoking							0.802			
	Current or Ex-smoker		25(20.2%)		7(23.3%)					
	Never		99(79.8%)		23(76.7%)					
Hypertension							0.419			
	Yes		61(49.2%)		13(43.3%)					
	No		63(50.8%)		17(56.7%)					
Ischemic heart disease							0.966			
	Yes		8(6.5%)		2(6.7%)					
	No		116(93.5%)		28(93.3%)					
Stroke							0.84			
	Yes		14(11.3%)		3(10.0%)					
	No		110(88.7%)		27(90.0%)					
Chronic renal failure							<0.01			
	Yes		11(8.9%)		11(36.7%)					
	No		113(91.1%)		19(63.3%)					
Premorbid ambulation state							<0.01	1.84	1.26	2.68
	Independent		80(64.5%)		8(26.7%)					
	Walking with aid		19(15.3%)		10(33.3%)					
	Wheel chair		17(13.7%)		7(23.3%)					
	Bedridden		8(6.5%)		5(16.7%)					
Preoperative laboratory finding										
	Hb (g/dl)			10.55 (2.3)		9.42 (1.4)	<0.01			
	WBC (/mm^3^)			10471 (5535)		12475 (5263)	<0.01			
	CRP (mg/dl)			6.16 (7.5)		10.62 (5.3)	<0.01			
	Creatinine (mg/dl)			1.82 (2.3)		2.82 (2.6)	0.251			
	HbA1C (%)			7.62 (1.9)		7.53 (1.9)	0.11			
Preoperative radiologic findings										
	X-ray									
	Osteomyelitis		18(14.5%)		7(23.3%)		0.24			
	None		106(85.5%)		23(76.7%)					
	CT angiogram*									
	Damaged vessel^†^						<0.01	4.38	2.360	8.151
	Number of damaged vessels^††^									
		None versus ≥ 1					0.133	2.903	0.619	13.615
		≤1 versus ≥ 2					<0.01	16.677	4.980	55.846
		≤2 versus 3					<0.01	21.583	6.483	71.86
	Anterior tibial artery						<0.01	10.405	4.088	26.486
		Patent								
		Stenosis > 50%, Diffuse atheromatosis								
		Occlusion								
	Posterior tibial artery						<0.01	5.062	2.633	9.733
		Patent								
		Stenosis > 50%, Diffuse atheromatosis								
		Occlusion								
	Peroneal artery						<0.01	4.229	2.240	7.984
		Patent								
		Stenosis > 50%, Diffuse atheromatosis								
		Occlusion								

*Statistical analysis was performed by univariate logistic regression analysis.

^†^Comparison of the distribution of the number of damaged vessels between limb salvage success group and failure group.

^††^Comparison of the number of damaged vessels between limb salvage success group and failure group.

The number of damaged vessels was defined as those with partial occlusion plus those with total occlusion.

Hb; hemoglobin, WBC; white blood cell, CRP; C-reactive protein, HbA1c; glycosylated hemoglobin, CT; computed tomography.

### Follow-up period and survival rate

**Figure 1:**
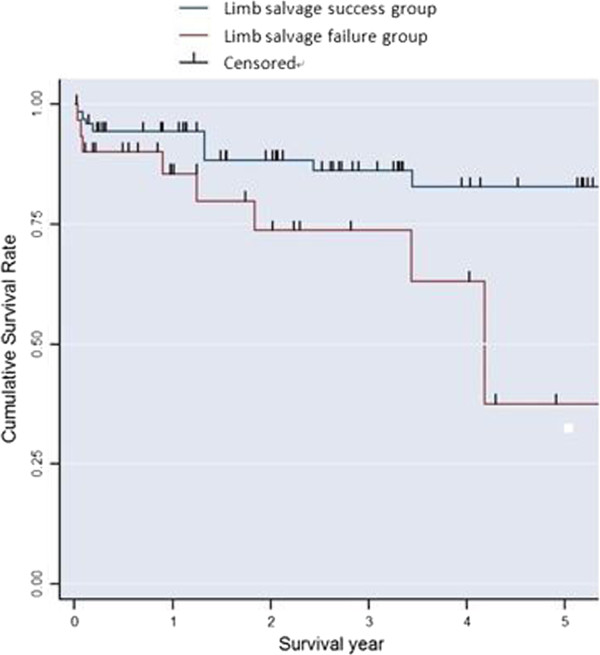
**Kaplan-Meier survival estimate for both groups.**

The average follow-up period was 118 weeks. The six-month survival rate was 91.9% for the success group and 76.7% for the failure group without statistical significance (Table 3). The Kaplan-Meier survival estimate was calculated for the limb salvage success group and failure group patients. A comparison between the two groups was performed with the log-rank test. The five-year survival rate was 81.6% for the limb salvage success group and 36.4% for the limb salvage failure group (P < 0.05) (Figure 1).

**Table 3:** **Survival rates**

Factors	Outcome		
	Limb salvage success group	Limb salvage failure group	P-value
	n (%)	n (%)	
Survived at 6 months			0.142
Yes	114 (91.9%)	23 (76.7%)	
No	10 (8.1%)	7 (23.3%)

## Discussion

The objective of this study was to identify any predictive factors of limb salvage success for patients with diabetic foot complications. Many studies have focused on the risk factors of diabetic foot ulceration and independent causation of multiple potential etiologic agents. However, no published studies have examined the risk factors for major amputation after limb-salvage surgery. Risk factors are important in predicting the prognosis of ulceration, yet many patients already have intractable ulceration prior to hospital admission. As a result, these studies are less helpful for the prognosis of patients in need of surgery for complicated diabetic foot [15, 16]. This study differs from previous studies in that it suggests the clinical predictors of limb salvage surgery failure. In this study, Hb, WBC, and CRP were risk factors of limb salvage surgery, but HbA1c was not. This is not surprising, given that Hb, WBC, and CRP are risk factors for diabetic foot complication, and hence a reflection of the patient’s general condition and the degree of wound inflammation [16]. On the other hand, unlike our findings showing no significant relationship between HbA1c and limb salvage surgery outcomes, previous studies have reported that HbA1c is a risk factor for diabetic foot complications [17–19]. Chronic renal failure is also an important risk factor for proximal osteotomy. In our study, 11 of 22 patients with chronic renal failure experienced limb salvage surgery failure. In previous reports, among chronic renal failure patients on dialysis who underwent limb salvage surgery, about 50% experienced failure and went to amputation. It has been reported that the risk of lower limb amputation is greater in diabetic foot patients with kidney disease [15, 20]. However, in this study, creatinine was not a significant risk factor. Why creatinine was not found to be a risk factor for salvage failure in our study, although it has been identified as a risk factor for major limb amputation in previous studies, cannot be explained satisfactorily. The authors supposed that the reason was that creatinine levels could be controlled directly depending on the treatment for renal failure, such as dialysis [15, 21].

To reflect the uniqueness of the patients with diabetic foot complications, a simple analysis method, the ambulation state, was used in this study. This can be relatively easily measured through a simple conversation with the patient. We found that the higher the ambulation state prior to surgery, the more successful the limb salvage operation. In other words, the postoperative walking ability was proportional to one’s walking ability before the surgery. CT angiogram was used to identify the status of the blood vessels prior to surgery and has been proven effective in prior studies [22–24]. Nevertheless, no studies have examined the failure of limb salvage surgery using the results of CT angiography until now. In the results of this study, the greater the number of damaged vessels as shown on CT angiogram, the greater was the difference of the odds ratio between the two groups. When comparing each blood vessel, a reduced vascular patency was found to be associated with failure. The number of normal blood vessels and the condition of each of the blood vessels had an effect on the results. In addition, it is worth considering that the diameter of the blood vessels of the lower limb is associated with the clinical outcomes of limb salvage surgery. The vessel diameter and odds ratio are largest in the anterior tibial artery, followed by the posterior tibial artery, and last, the peroneal artery [25].

Stone et al. [20] reported the 1-, 3- and 5-year survival rates of diabetic foot patients undergoing transmetatarsal amputation to be 73%, 68%, and 62%, respectively. In our study, the 5-year survival rate of the limb salvage group was 81.6%. This may be higher because we included a high proportion of patients with toe amputation. We also found that in our success and failure groups, the 6-month survival rates showed no statistically significant difference, but the 5-year survival rate of the limb salvage surgery success group was significantly higher, meaning that the patient’s age and life expectancy may help guide further surgical treatment.

## Conclusions

This study evaluated the factors predictive of the success of limb salvage surgery and identified indicators for preserving the limbs of patients with diabetic foot complications, allowing the establishment of an appropriate amputation level of the diabetic foot and minimizing subsequent operations.
